# On the Supposed Binoxide of Protein

**Published:** 1846-04

**Authors:** Baron Liebig


### RECORD OF MEDICAL SCIENCE.

*On the supposed Binoxide of Protein.* By BARON LIEBIG.—According to Bouchardat, if moist fibrine be immersed in water acidulated with a two-thousandth part of hydrochloric acid, the fibrine speedily becomes gelatinous, and on continuing the action of the dilute hydrochloric acid, a solution is obtained, which is turbid. This turbidity appears to arise from a slight admixture of a fatty substance. That portion of the fibrine which is thus soluble in hydrochloric acid, Bouchardat designates by the term ALBUMINOSE, the insoluble part by that of EPIDERMOSE.

Mulder states that this experiment was repeated by Herrn Von Baumhauer. The solution of fibrine in dilute hydrochloric acid Baumhauer precipitated by means of carbonate of ammonia, and treated the precipitate with alcohol. After drying, the precipitate yielded on analysis,

| | 1. | 2. |
|---|---|---|
| Carbon, | 53.64 | 55.62 |
| Hydrogen, | 6.88 | 6.73 |
| Nitrogen, | 15.88 | — |
| Oxygen, | 23.64 | — |

Mr. Mulder concludes, from these experiments, that "the albuminose is a product of the oxidation of protein, which, no doubt, is formed by the action of the atmospheric oxygen upon the matter formed and dissolved by the hydrochloric acid. It is *binoxide* of protein$=C_{40}H_{62}N_{10}O_{14}$."

We have of late been so overwhelmed with descriptions of substances, which, although greatly differing in their properties, yet, according to their analyses, must be considered as oxides of protein, that I was very desirous of convincing myself, by experiment, of the existence of at least one of them.

I have found, that if we are to understand by the designation, *binoxide of protein*, a substance which contains no sulphur, the substance investigated by Baumhauer cannot belong to such a class of bodies as oxides of protein, since the whole amount of sulphur present in fibrine exists unaltered in this new substance.

In the first place, I observed that the atmosphere and its oxygen have no share in the solution of fibrine in hydrochloric acid. If moist fibrine is allowed to become gelatinous in dilute hydrochloric acid, and the mixture copiously diluted with water, and immediately afterwards filtered, before the solution has taken place, the presence of lime and potass may be detected in the filtered fluid. If the fluid be exposed to the protracted action of a higher degree of heat so as to effect the solution, and a portion of this heated to boiling, with an excess of potass, the subsequent neutralization with acetic acid gives rise to a copious evolution of sulphuretted hydrogen, and a drop of acetate of lead produces in it a black precipitate. Upon solution of fibrine in dilute hydrochloric acid of the above given strength, therefore, the sulphur remains combined with the other elements of the fibrine. If this solution of fibrine in hydrochloric acid (the albuminose of Bouchardat) is mixed with a solution of common salt, sulphate of soda, or nitrate of potass, the mixture coagulates, forming a caseous or albuminous mass, which may be washed with a solution of common salt, the fluid which runs off, containing common salt, contains no longer any perceptible traces of lime or phosphoric acid: these two substances are, however, contained in the precipitate. The fluid contains no oxygen compound of sulphur, and gives no re-action of sulphuret of potassium when boiled with potass ley. But, according Mulder, the albuminose of Bouchardat is binoxide of protein; were it so, the precipitate ought to contain neither phosphate of lime nor sulphur; but on dissolving it in potass ley, and boiling, and adding, subsequently, salts of lead, a black precipitate of sulphuret of lead is produced, and the action of acid produces an evolution of sulphuretted hydrogen.

This substance is, therefore, not a binoxide of protein.—*London Lancet.*

